# 9-(4-Bromo­phen­yl)-3,6-di-*tert*-butyl-9*H*-carbazole

**DOI:** 10.1107/S1600536811047386

**Published:** 2011-11-16

**Authors:** Jing-Ya Zhang, Wei-Yi Zhang

**Affiliations:** a Pharmacy College, Henan University of Traditional Chinese Medicine, Zhengzhou 450008, People’s Republic of China; b Department of Obstetrics and Gynecology, The First Affiliated Hospital of Henan University of Traditional Chinese Medicine, Zhengzhou 450003, People’s Republic of China

## Abstract

The asymmetric unit of the title compound, C_26_H_28_BrN, contains two independent mol­ecules in which the carbazole rings are almost planar, with r.m.s. deviations of 0.0212 (1) and 0.0229 (1) Å. The dihedral angles between the carbazole ring system and the pendent benzene ring are 60.5 (1) and 56.3 (1)° in the two mol­ecules. In the crystal, mol­ecules are linked into chains along the *b* axis by C—H⋯π inter­actions.

## Related literature

For background to the applications of the title compound, see: Wang *et al.* (2008 ▶). For the synthesis of the title compound, see: Weber *et al.* (2011 ▶). For bond-length data, see: Allen *et al.* (1987 ▶).
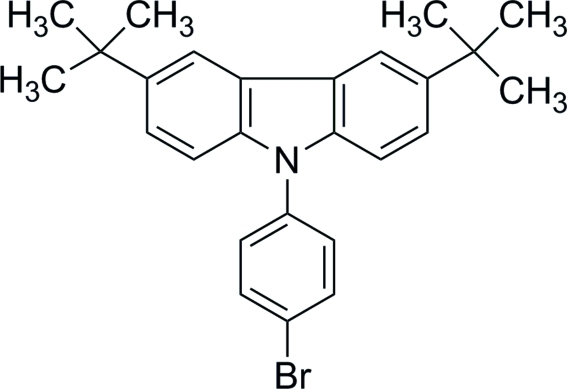


## Experimental

### 

#### Crystal data

C_26_H_28_BrN
*M* *_r_* = 434.40
Triclinic, 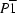

*a* = 5.9300 (12) Å
*b* = 17.634 (4) Å
*c* = 22.343 (5) Å
α = 100.38 (3)°
β = 95.13 (3)°
γ = 99.32 (3)°
*V* = 2250.8 (8) Å^3^
*Z* = 4
Mo *K*α radiation
μ = 1.84 mm^−1^
*T* = 293 K
0.20 × 0.10 × 0.10 mm

#### Data collection

Enraf–Nonius CAD-4 diffractometer
Absorption correction: ψ scan (North *et al.*, 1968 ▶) *T*_min_ = 0.710, *T*_max_ = 0.838
9130 measured reflections
8267 independent reflections
3724 reflections with *I* > 2σ(*I*)
*R*_int_ = 0.054
3 standard reflections every 200 reflections  intensity decay: 1%

#### Refinement

*R*[*F* ^2^ > 2σ(*F* ^2^)] = 0.076
*wR*(*F* ^2^) = 0.160
*S* = 1.00
8267 reflections
505 parameters
H-atom parameters constrained
Δρ_max_ = 0.52 e Å^−3^
Δρ_min_ = −0.54 e Å^−3^

### 

Data collection: *CAD-4 Software* (Enraf–Nonius, 1985 ▶); cell refinement: *CAD-4 Software*; data reduction: *XCAD4* (Harms & Wocadlo,1995 ▶); program(s) used to solve structure: *SHELXS97* (Sheldrick, 2008 ▶); program(s) used to refine structure: *SHELXL97* (Sheldrick, 2008 ▶); molecular graphics: *SHELXTL* (Sheldrick, 2008 ▶); software used to prepare material for publication: *SHELXTL*.

## Supplementary Material

Crystal structure: contains datablock(s) I, global. DOI: 10.1107/S1600536811047386/vm2131sup1.cif

Structure factors: contains datablock(s) I. DOI: 10.1107/S1600536811047386/vm2131Isup2.hkl

Supplementary material file. DOI: 10.1107/S1600536811047386/vm2131Isup3.cml

Additional supplementary materials:  crystallographic information; 3D view; checkCIF report

## Figures and Tables

**Table 1:** Hydrogen-bond geometry (Å, °) *Cg*3, *Cg*11 and *Cg*8 are the centroids of the C7–C12, C47–C52 and N2/C27/C32–C34 rings, respectively.

*D*—H⋯*A*	*D*—H	H⋯*A*	*D*⋯*A*	*D*—H⋯*A*
C22—H22*A*⋯*Cg*3^i^	0.93	2.75	3.544 (8)	144
C25—H25*A*⋯*Cg*11^ii^	0.93	2.92	3.511 (7)	123
C52—H52*A*⋯*Cg*8^iii^	0.93	2.95	3.591 (8)	127

Symmetry codes: (i) 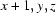; (ii) 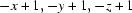; (iii) 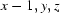.

**Fig. 1.**
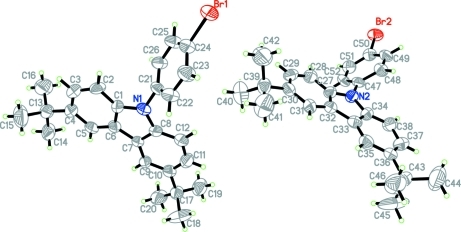
Molecular structure of (I) with the atom-numbering scheme and displacement ellipsoids drawn at the 50% probability level.

**Fig. 2.**
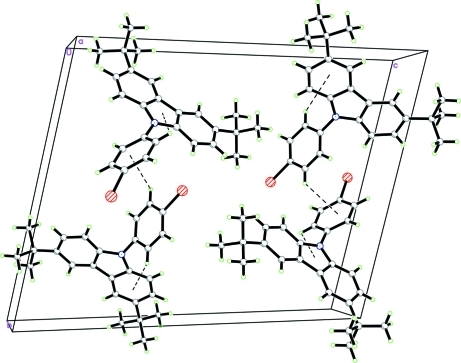
Packing diagram of (I) showing C—H···π interactions as dashed lines.

## supplementary crystallographic information

### Comment

The title compound, 9-(4-bromophenyl)-3,6-di-*tert*-butyl-9*H*-carbazole, is an important intermediate, which can be utilized to synthesize organic semiconductors and conjugated polymers (Wang *et al.* , 2008). Here we report here its crystal structure (Fig. 1).

The two molecules in the asymmetric unit have the same conformation (r.m.s. deviation 0.2092 Å for all non-H atoms fitted) . The bond lengths and angles are within normal ranges (Allen *et al.*, 1987). The carbazole rings are almost planar. The dihedral angles of the rings A(C1—C6/N1/C7—C12), B(C21—C26), C(C27—C32/N2/C33—C38), D(C47—C52) are: A/B = 60.5 (1)°, C/D = 56.3 (1)°.

In the crystal packing, there are not classic hydrogen bonds found. The molecular chains are linked by C—H···π interactions (Table 1) to give a three-dimensional network, which seems to be very effective in the stabilization of the crystal structure.

### Experimental

The title compound, (I) was prepared by a method reported in literature (Weber *et al.* , 2011). The crystals were obtained by dissolving (I) (0.5 g) in methanol (50 ml) and evaporating the solvent slowly at room temperature for about 10 d.

### Refinement

Aromatic H atoms were positioned geometrically with C—H = 0.93 Å, and constrained to ride on their parent atoms, with *U*_iso_(H) = 1.2*U*_eq_(C). Other H atoms were positioned geometrically and refined using a riding model, with C—H = 0.97 Å for alkyl H, *U*_iso_(H) = *1.5U*_eq_(C).

### Crystal data

**Table:**

C_26_H_28_BrN	*Z* = 4
*M**_r_* = 434.40	*F*(000) = 904
Triclinic, *P*1	*D*_x_ = 1.282 Mg m^−^^3^
Hall symbol: -P 1	Mo *K*α radiation, λ = 0.71073 Å
*a* = 5.9300 (12) Å	Cell parameters from 25 reflections
*b* = 17.634 (4) Å	θ = 10–13°
*c* = 22.343 (5) Å	µ = 1.84 mm^−^^1^
α = 100.38 (3)°	*T* = 293 K
β = 95.13 (3)°	Block, colourless
γ = 99.32 (3)°	0.20 × 0.10 × 0.10 mm
*V* = 2250.8 (8) Å^3^	

### Data collection

**Table:**

Enraf–Nonius CAD-4 diffractometer	3724 reflections with *I* > 2σ(*I*)
Radiation source: fine-focus sealed tube	*R*_int_ = 0.054
graphite	θ_max_ = 25.4°, θ_min_ = 1.2°
ω/2θ scans	*h* = 0→7
Absorption correction: ψ scan (North *et al.*, 1968)	*k* = −21→20
*T*_min_ = 0.710, *T*_max_ = 0.838	*l* = −26→26
9130 measured reflections	3 standard reflections every 200 reflections
8267 independent reflections	intensity decay: 1%

### Refinement

**Table:**

Refinement on *F*^2^	Primary atom site location: structure-invariant direct methods
Least-squares matrix: full	Secondary atom site location: difference Fourier map
*R*[*F*^2^ > 2σ(*F*^2^)] = 0.076	Hydrogen site location: inferred from neighbouring sites
*wR*(*F*^2^) = 0.160	H-atom parameters constrained
*S* = 1.00	*w* = 1/[σ^2^(*F*_o_^2^) + (0.059*P*)^2^] where *P* = (*F*_o_^2^ + 2*F*_c_^2^)/3
8267 reflections	(Δ/σ)_max_ < 0.001
505 parameters	Δρ_max_ = 0.52 e Å^−^^3^
0 restraints	Δρ_min_ = −0.54 e Å^−^^3^

### Special details

**Table:**

Geometry. All e.s.d.'s (except the e.s.d. in the dihedral angle between two l.s. planes) are estimated using the full covariance matrix. The cell e.s.d.'s are taken into account individually in the estimation of e.s.d.'s in distances, angles and torsion angles; correlations between e.s.d.'s in cell parameters are only used when they are defined by crystal symmetry. An approximate (isotropic) treatment of cell e.s.d.'s is used for estimating e.s.d.'s involving l.s. planes.
Refinement. Refinement of *F*^2^ against ALL reflections. The weighted *R*-factor *wR* and goodness of fit *S* are based on *F*^2^, conventional *R*-factors *R* are based on *F*, with *F* set to zero for negative *F*^2^. The threshold expression of *F*^2^ > σ(*F*^2^) is used only for calculating *R*-factors(gt) *etc*. and is not relevant to the choice of reflections for refinement. *R*-factors based on *F*^2^ are statistically about twice as large as those based on *F*, and *R*- factors based on ALL data will be even larger.

### Fractional atomic coordinates and isotropic or equivalent isotropic displacement parameters (Å^2^)

**Table:**

	*x*	*y*	*z*	*U*_iso_*/*U*_eq_	
Br1	1.18728 (13)	0.51528 (4)	0.36422 (3)	0.0683 (3)	
N1	0.6647 (9)	0.7449 (3)	0.2618 (2)	0.0568 (15)	
C1	0.5559 (11)	0.7335 (3)	0.2012 (3)	0.0503 (17)	
C2	0.5832 (11)	0.6807 (3)	0.1511 (3)	0.0556 (18)	
H2A	0.6799	0.6445	0.1535	0.067*	
C3	0.4579 (13)	0.6840 (4)	0.0960 (3)	0.066 (2)	
H3A	0.4754	0.6491	0.0609	0.079*	
C4	0.3087 (11)	0.7362 (4)	0.0903 (3)	0.0546 (17)	
C5	0.2891 (11)	0.7887 (3)	0.1427 (3)	0.0549 (18)	
H5A	0.1913	0.8246	0.1405	0.066*	
C6	0.4127 (10)	0.7888 (3)	0.1984 (3)	0.0472 (16)	
C7	0.4345 (11)	0.8367 (3)	0.2589 (3)	0.0505 (17)	
C8	0.5856 (11)	0.8078 (3)	0.2972 (3)	0.0523 (17)	
C9	0.3366 (11)	0.9001 (3)	0.2840 (3)	0.0480 (16)	
H9A	0.2386	0.9210	0.2592	0.058*	
C10	0.3866 (12)	0.9320 (4)	0.3466 (3)	0.0553 (18)	
C11	0.5337 (13)	0.8995 (4)	0.3825 (3)	0.070 (2)	
H11A	0.5652	0.9209	0.4242	0.084*	
C12	0.6350 (12)	0.8375 (4)	0.3596 (3)	0.0610 (19)	
H12A	0.7317	0.8165	0.3847	0.073*	
C13	0.1759 (12)	0.7339 (4)	0.0287 (3)	0.0584 (18)	
C14	0.0161 (13)	0.7946 (4)	0.0318 (3)	0.084 (3)	
H14A	−0.0921	0.7849	0.0602	0.127*	
H14B	−0.0654	0.7905	−0.0081	0.127*	
H14C	0.1061	0.8463	0.0451	0.127*	
C15	0.3463 (15)	0.7520 (5)	−0.0174 (4)	0.113 (3)	
H15A	0.4463	0.7142	−0.0212	0.169*	
H15B	0.4363	0.8036	−0.0031	0.169*	
H15C	0.2621	0.7496	−0.0567	0.169*	
C16	0.0283 (15)	0.6533 (4)	0.0045 (4)	0.103 (3)	
H16A	−0.0786	0.6419	0.0329	0.154*	
H16B	0.1253	0.6145	−0.0001	0.154*	
H16C	−0.0548	0.6527	−0.0345	0.154*	
C17	0.2911 (12)	1.0038 (4)	0.3755 (3)	0.0586 (18)	
C18	0.4896 (15)	1.0737 (4)	0.3912 (4)	0.119 (3)	
H18A	0.5515	1.0830	0.3544	0.178*	
H18B	0.6077	1.0628	0.4190	0.178*	
H18C	0.4344	1.1194	0.4102	0.178*	
C19	0.1969 (14)	0.9909 (4)	0.4331 (3)	0.094 (3)	
H19A	0.1380	1.0363	0.4509	0.140*	
H19B	0.3169	0.9822	0.4615	0.140*	
H19C	0.0750	0.9460	0.4242	0.140*	
C20	0.0997 (14)	1.0215 (4)	0.3337 (3)	0.093 (3)	
H20A	0.1563	1.0310	0.2963	0.139*	
H20B	0.0469	1.0671	0.3537	0.139*	
H20C	−0.0255	0.9776	0.3247	0.139*	
C21	0.7872 (12)	0.6911 (4)	0.2852 (3)	0.0483 (16)	
C22	1.0060 (13)	0.7177 (4)	0.3155 (3)	0.0626 (19)	
H22A	1.0738	0.7700	0.3194	0.075*	
C23	1.1281 (12)	0.6664 (4)	0.3407 (3)	0.065 (2)	
H23A	1.2742	0.6842	0.3622	0.078*	
C24	1.0248 (12)	0.5896 (4)	0.3323 (3)	0.0550 (18)	
C25	0.8063 (12)	0.5632 (4)	0.3026 (3)	0.0608 (19)	
H25A	0.7385	0.5108	0.2980	0.073*	
C26	0.6872 (12)	0.6147 (3)	0.2794 (3)	0.0554 (18)	
H26A	0.5379	0.5971	0.2598	0.066*	
Br2	−0.10255 (13)	0.46827 (4)	0.85902 (4)	0.0709 (3)	
N2	0.5597 (9)	0.7103 (3)	0.7642 (2)	0.0541 (14)	
C27	0.5939 (11)	0.6989 (3)	0.7025 (3)	0.0484 (16)	
C28	0.4698 (12)	0.6432 (4)	0.6522 (3)	0.0607 (19)	
H28A	0.3398	0.6084	0.6568	0.073*	
C29	0.5478 (12)	0.6425 (4)	0.5968 (3)	0.0621 (19)	
H29A	0.4670	0.6062	0.5633	0.074*	
C30	0.7458 (12)	0.6941 (4)	0.5872 (3)	0.0561 (18)	
C31	0.8639 (12)	0.7489 (4)	0.6379 (3)	0.0591 (18)	
H31A	0.9930	0.7842	0.6335	0.071*	
C32	0.7872 (11)	0.7503 (3)	0.6948 (3)	0.0506 (17)	
C33	0.8728 (11)	0.7983 (4)	0.7553 (3)	0.0508 (17)	
C34	0.7351 (12)	0.7715 (4)	0.7960 (3)	0.0552 (18)	
C35	1.0543 (12)	0.8605 (4)	0.7758 (3)	0.0601 (19)	
H35A	1.1509	0.8773	0.7484	0.072*	
C36	1.0928 (13)	0.8978 (4)	0.8369 (3)	0.0594 (19)	
C37	0.9450 (13)	0.8687 (4)	0.8764 (3)	0.067 (2)	
H37A	0.9663	0.8937	0.9174	0.080*	
C38	0.7728 (13)	0.8061 (4)	0.8576 (3)	0.067 (2)	
H38A	0.6822	0.7869	0.8854	0.081*	
C39	0.8219 (12)	0.6878 (4)	0.5232 (3)	0.0601 (19)	
C40	0.6346 (14)	0.7003 (5)	0.4787 (3)	0.102 (3)	
H40A	0.6860	0.6975	0.4390	0.154*	
H40B	0.5961	0.7510	0.4922	0.154*	
H40C	0.5011	0.6605	0.4764	0.154*	
C41	1.0313 (14)	0.7502 (5)	0.5216 (4)	0.109 (3)	
H41A	1.0722	0.7449	0.4807	0.163*	
H41B	1.1582	0.7436	0.5488	0.163*	
H41C	0.9950	0.8013	0.5344	0.163*	
C42	0.8816 (17)	0.6065 (5)	0.5030 (4)	0.122 (4)	
H42A	0.9307	0.6022	0.4629	0.183*	
H42B	0.7483	0.5671	0.5019	0.183*	
H42C	1.0034	0.5992	0.5315	0.183*	
C43	1.2806 (14)	0.9709 (4)	0.8600 (3)	0.072 (2)	
C44	1.3953 (19)	0.9693 (6)	0.9218 (4)	0.159 (5)	
H44A	1.4664	0.9238	0.9195	0.239*	
H44B	1.2829	0.9674	0.9501	0.239*	
H44C	1.5105	1.0157	0.9357	0.239*	
C45	1.1659 (15)	1.0427 (4)	0.8613 (5)	0.149 (5)	
H45A	1.2799	1.0895	0.8749	0.223*	
H45B	1.0508	1.0414	0.8888	0.223*	
H45C	1.0951	1.0424	0.8208	0.223*	
C46	1.4614 (15)	0.9776 (5)	0.8171 (4)	0.106 (3)	
H46A	1.5384	0.9335	0.8148	0.158*	
H46B	1.5713	1.0251	0.8320	0.158*	
H46C	1.3897	0.9786	0.7770	0.158*	
C47	0.4023 (13)	0.6594 (3)	0.7889 (3)	0.0512 (17)	
C48	0.4838 (12)	0.6231 (4)	0.8355 (3)	0.0593 (18)	
H48A	0.6383	0.6354	0.8516	0.071*	
C49	0.3324 (13)	0.5688 (3)	0.8573 (3)	0.0571 (18)	
H49A	0.3858	0.5444	0.8879	0.069*	
C50	0.1026 (12)	0.5510 (4)	0.8337 (3)	0.0575 (18)	
C51	0.0264 (13)	0.5897 (4)	0.7890 (3)	0.0636 (19)	
H51A	−0.1283	0.5786	0.7731	0.076*	
C52	0.1742 (12)	0.6433 (4)	0.7683 (3)	0.0565 (18)	
H52A	0.1179	0.6696	0.7393	0.068*	

### Atomic displacement parameters (Å^2^)

**Table:**

	*U*^11^	*U*^22^	*U*^33^	*U*^12^	*U*^13^	*U*^23^
Br1	0.0855 (6)	0.0567 (5)	0.0698 (5)	0.0332 (4)	−0.0015 (4)	0.0187 (4)
N1	0.079 (4)	0.041 (3)	0.057 (4)	0.024 (3)	0.007 (3)	0.017 (3)
C1	0.068 (5)	0.046 (4)	0.038 (4)	0.017 (4)	0.002 (3)	0.008 (3)
C2	0.064 (5)	0.039 (4)	0.064 (5)	0.025 (3)	0.004 (4)	0.001 (3)
C3	0.090 (6)	0.053 (4)	0.054 (5)	0.019 (4)	0.014 (4)	−0.001 (4)
C4	0.065 (5)	0.051 (4)	0.050 (4)	0.016 (4)	0.010 (4)	0.008 (3)
C5	0.072 (5)	0.042 (4)	0.058 (5)	0.021 (4)	0.008 (4)	0.020 (3)
C6	0.056 (4)	0.042 (4)	0.049 (4)	0.019 (3)	0.011 (3)	0.013 (3)
C7	0.079 (5)	0.034 (3)	0.044 (4)	0.017 (3)	0.012 (4)	0.012 (3)
C8	0.070 (5)	0.035 (4)	0.051 (4)	0.012 (3)	−0.002 (4)	0.009 (3)
C9	0.060 (5)	0.043 (4)	0.044 (4)	0.019 (3)	0.010 (3)	0.007 (3)
C10	0.069 (5)	0.045 (4)	0.056 (5)	0.016 (4)	0.013 (4)	0.013 (3)
C11	0.092 (6)	0.062 (5)	0.053 (5)	0.026 (5)	−0.004 (4)	0.001 (4)
C12	0.083 (6)	0.044 (4)	0.058 (5)	0.018 (4)	−0.001 (4)	0.016 (4)
C13	0.061 (5)	0.057 (4)	0.052 (4)	0.006 (4)	−0.004 (4)	0.006 (3)
C14	0.116 (7)	0.094 (6)	0.049 (5)	0.046 (5)	−0.011 (4)	0.015 (4)
C15	0.132 (8)	0.147 (9)	0.080 (6)	0.048 (7)	0.047 (6)	0.041 (6)
C16	0.122 (8)	0.077 (6)	0.089 (6)	−0.007 (5)	−0.024 (6)	0.003 (5)
C17	0.067 (5)	0.054 (4)	0.057 (5)	0.025 (4)	0.005 (4)	0.005 (4)
C18	0.108 (8)	0.050 (5)	0.176 (10)	0.006 (5)	0.024 (7)	−0.029 (5)
C19	0.111 (7)	0.101 (6)	0.070 (6)	0.038 (6)	0.013 (5)	0.002 (5)
C20	0.120 (7)	0.068 (5)	0.094 (6)	0.051 (5)	0.005 (6)	−0.001 (4)
C21	0.055 (5)	0.043 (4)	0.050 (4)	0.013 (4)	−0.003 (3)	0.019 (3)
C22	0.073 (6)	0.041 (4)	0.077 (5)	0.012 (4)	0.008 (4)	0.018 (4)
C23	0.063 (5)	0.057 (5)	0.075 (5)	0.010 (4)	−0.005 (4)	0.019 (4)
C24	0.063 (5)	0.044 (4)	0.061 (4)	0.009 (4)	0.008 (4)	0.017 (3)
C25	0.062 (5)	0.042 (4)	0.083 (5)	0.018 (4)	0.005 (4)	0.019 (4)
C26	0.061 (5)	0.039 (4)	0.066 (5)	0.011 (4)	0.004 (4)	0.010 (3)
Br2	0.0782 (6)	0.0519 (5)	0.0866 (6)	0.0015 (4)	0.0305 (4)	0.0237 (4)
N2	0.065 (4)	0.045 (3)	0.048 (3)	−0.007 (3)	0.009 (3)	0.011 (3)
C27	0.054 (5)	0.039 (4)	0.050 (4)	0.002 (3)	0.007 (4)	0.009 (3)
C28	0.067 (5)	0.054 (4)	0.059 (5)	0.004 (4)	0.008 (4)	0.013 (4)
C29	0.080 (6)	0.042 (4)	0.062 (5)	0.011 (4)	0.011 (4)	0.002 (3)
C30	0.074 (5)	0.053 (4)	0.047 (4)	0.018 (4)	0.011 (4)	0.020 (4)
C31	0.075 (5)	0.055 (4)	0.048 (4)	0.006 (4)	0.003 (4)	0.018 (4)
C32	0.062 (5)	0.043 (4)	0.049 (4)	0.013 (4)	0.005 (4)	0.012 (3)
C33	0.053 (5)	0.043 (4)	0.060 (5)	0.007 (4)	0.013 (4)	0.017 (4)
C34	0.068 (5)	0.046 (4)	0.046 (4)	0.001 (4)	0.000 (4)	0.008 (3)
C35	0.065 (5)	0.058 (4)	0.060 (5)	0.006 (4)	0.008 (4)	0.023 (4)
C36	0.080 (6)	0.039 (4)	0.057 (5)	0.007 (4)	−0.007 (4)	0.017 (4)
C37	0.076 (6)	0.048 (4)	0.071 (5)	0.005 (4)	0.006 (4)	0.005 (4)
C38	0.093 (6)	0.055 (4)	0.052 (5)	0.004 (4)	0.016 (4)	0.011 (4)
C39	0.064 (5)	0.072 (5)	0.048 (4)	0.025 (4)	0.009 (4)	0.008 (4)
C40	0.087 (7)	0.153 (8)	0.075 (6)	0.030 (6)	0.009 (5)	0.035 (6)
C41	0.093 (7)	0.136 (8)	0.089 (6)	−0.008 (6)	0.030 (5)	0.018 (6)
C42	0.193 (11)	0.110 (7)	0.090 (7)	0.079 (7)	0.053 (7)	0.026 (6)
C43	0.084 (6)	0.058 (5)	0.060 (5)	−0.006 (4)	−0.007 (4)	0.003 (4)
C44	0.190 (12)	0.152 (10)	0.096 (8)	−0.060 (8)	−0.053 (8)	0.035 (7)
C45	0.103 (8)	0.039 (5)	0.284 (14)	−0.003 (5)	0.020 (9)	−0.004 (7)
C46	0.107 (7)	0.095 (7)	0.101 (7)	−0.024 (5)	0.012 (6)	0.020 (5)
C47	0.071 (5)	0.036 (4)	0.053 (4)	0.021 (4)	0.018 (4)	0.014 (3)
C48	0.061 (5)	0.062 (5)	0.062 (5)	0.022 (4)	0.016 (4)	0.017 (4)
C49	0.072 (6)	0.048 (4)	0.060 (5)	0.017 (4)	0.017 (4)	0.023 (3)
C50	0.056 (5)	0.061 (5)	0.058 (5)	0.018 (4)	0.021 (4)	0.006 (4)
C51	0.070 (5)	0.063 (5)	0.061 (5)	0.011 (4)	0.022 (4)	0.015 (4)
C52	0.059 (5)	0.055 (4)	0.065 (5)	0.026 (4)	0.007 (4)	0.024 (4)

### Geometric parameters (Å, °)

**Table:**

Br1—C24	1.944 (6)	Br2—C50	1.944 (7)
N1—C1	1.411 (7)	N2—C27	1.395 (7)
N1—C8	1.413 (7)	N2—C47	1.405 (7)
N1—C21	1.427 (7)	N2—C34	1.407 (7)
C1—C2	1.361 (8)	C27—C32	1.386 (8)
C1—C6	1.398 (7)	C27—C28	1.411 (8)
C2—C3	1.396 (8)	C28—C29	1.357 (8)
C2—H2A	0.9300	C28—H28A	0.9300
C3—C4	1.391 (8)	C29—C30	1.422 (9)
C3—H3A	0.9300	C29—H29A	0.9300
C4—C5	1.382 (8)	C30—C31	1.397 (8)
C4—C13	1.514 (8)	C30—C39	1.528 (8)
C5—C6	1.387 (8)	C31—C32	1.387 (8)
C5—H5A	0.9300	C31—H31A	0.9300
C6—C7	1.441 (8)	C32—C33	1.457 (8)
C7—C9	1.394 (7)	C33—C34	1.372 (8)
C7—C8	1.402 (8)	C33—C35	1.385 (8)
C8—C12	1.385 (8)	C34—C38	1.383 (8)
C9—C10	1.398 (8)	C35—C36	1.386 (8)
C9—H9A	0.9300	C35—H35A	0.9300
C10—C11	1.391 (8)	C36—C37	1.402 (9)
C10—C17	1.530 (8)	C36—C43	1.536 (9)
C11—C12	1.372 (8)	C37—C38	1.352 (9)
C11—H11A	0.9300	C37—H37A	0.9300
C12—H12A	0.9300	C38—H38A	0.9300
C13—C16	1.521 (9)	C39—C40	1.494 (9)
C13—C14	1.536 (8)	C39—C41	1.527 (9)
C13—C15	1.544 (9)	C39—C42	1.531 (9)
C14—H14A	0.9600	C40—H40A	0.9600
C14—H14B	0.9600	C40—H40B	0.9600
C14—H14C	0.9600	C40—H40C	0.9600
C15—H15A	0.9600	C41—H41A	0.9600
C15—H15B	0.9600	C41—H41B	0.9600
C15—H15C	0.9600	C41—H41C	0.9600
C16—H16A	0.9600	C42—H42A	0.9600
C16—H16B	0.9600	C42—H42B	0.9600
C16—H16C	0.9600	C42—H42C	0.9600
C17—C19	1.487 (9)	C43—C44	1.491 (10)
C17—C20	1.513 (9)	C43—C46	1.505 (10)
C17—C18	1.526 (9)	C43—C45	1.530 (10)
C18—H18A	0.9600	C44—H44A	0.9600
C18—H18B	0.9600	C44—H44B	0.9600
C18—H18C	0.9600	C44—H44C	0.9600
C19—H19A	0.9600	C45—H45A	0.9600
C19—H19B	0.9600	C45—H45B	0.9600
C19—H19C	0.9600	C45—H45C	0.9600
C20—H20A	0.9600	C46—H46A	0.9600
C20—H20B	0.9600	C46—H46B	0.9600
C20—H20C	0.9600	C46—H46C	0.9600
C21—C26	1.359 (8)	C47—C52	1.356 (8)
C21—C22	1.376 (8)	C47—C48	1.406 (8)
C22—C23	1.407 (8)	C48—C49	1.390 (8)
C22—H22A	0.9300	C48—H48A	0.9300
C23—C24	1.365 (8)	C49—C50	1.381 (8)
C23—H23A	0.9300	C49—H49A	0.9300
C24—C25	1.370 (8)	C50—C51	1.388 (8)
C25—C26	1.379 (8)	C51—C52	1.356 (8)
C25—H25A	0.9300	C51—H51A	0.9300
C26—H26A	0.9300	C52—H52A	0.9300
			
C1—N1—C8	107.6 (5)	C27—N2—C47	124.0 (5)
C1—N1—C21	125.1 (5)	C27—N2—C34	107.1 (5)
C8—N1—C21	125.8 (5)	C47—N2—C34	128.0 (5)
C2—C1—C6	122.7 (6)	C32—C27—N2	109.9 (5)
C2—C1—N1	128.3 (6)	C32—C27—C28	120.5 (6)
C6—C1—N1	108.9 (5)	N2—C27—C28	129.5 (6)
C1—C2—C3	116.2 (6)	C29—C28—C27	117.3 (6)
C1—C2—H2A	121.9	C29—C28—H28A	121.3
C3—C2—H2A	121.9	C27—C28—H28A	121.3
C4—C3—C2	124.1 (6)	C28—C29—C30	123.8 (6)
C4—C3—H3A	118.0	C28—C29—H29A	118.1
C2—C3—H3A	118.0	C30—C29—H29A	118.1
C5—C4—C3	117.0 (6)	C31—C30—C29	117.5 (6)
C5—C4—C13	122.9 (6)	C31—C30—C39	122.7 (6)
C3—C4—C13	120.1 (6)	C29—C30—C39	119.8 (6)
C4—C5—C6	121.2 (6)	C32—C31—C30	119.5 (6)
C4—C5—H5A	119.4	C32—C31—H31A	120.2
C6—C5—H5A	119.4	C30—C31—H31A	120.2
C5—C6—C1	118.7 (6)	C27—C32—C31	121.3 (6)
C5—C6—C7	133.9 (6)	C27—C32—C33	106.1 (6)
C1—C6—C7	107.4 (5)	C31—C32—C33	132.5 (6)
C9—C7—C8	118.8 (6)	C34—C33—C35	119.9 (6)
C9—C7—C6	133.8 (6)	C34—C33—C32	107.4 (6)
C8—C7—C6	107.4 (5)	C35—C33—C32	132.7 (6)
C12—C8—C7	122.8 (6)	C33—C34—C38	121.0 (7)
C12—C8—N1	128.6 (6)	C33—C34—N2	109.4 (6)
C7—C8—N1	108.6 (5)	C38—C34—N2	129.6 (6)
C7—C9—C10	119.5 (6)	C33—C35—C36	120.4 (6)
C7—C9—H9A	120.2	C33—C35—H35A	119.8
C10—C9—H9A	120.2	C36—C35—H35A	119.8
C11—C10—C9	118.9 (6)	C35—C36—C37	117.3 (6)
C11—C10—C17	119.9 (6)	C35—C36—C43	121.6 (7)
C9—C10—C17	121.2 (6)	C37—C36—C43	121.0 (7)
C12—C11—C10	123.5 (6)	C38—C37—C36	123.1 (7)
C12—C11—H11A	118.2	C38—C37—H37A	118.5
C10—C11—H11A	118.2	C36—C37—H37A	118.5
C11—C12—C8	116.5 (6)	C37—C38—C34	118.2 (7)
C11—C12—H12A	121.8	C37—C38—H38A	120.9
C8—C12—H12A	121.8	C34—C38—H38A	120.9
C4—C13—C16	110.3 (6)	C40—C39—C41	106.4 (6)
C4—C13—C14	112.5 (5)	C40—C39—C30	110.4 (6)
C16—C13—C14	107.8 (6)	C41—C39—C30	112.0 (6)
C4—C13—C15	109.5 (6)	C40—C39—C42	110.0 (7)
C16—C13—C15	109.1 (6)	C41—C39—C42	109.1 (7)
C14—C13—C15	107.5 (6)	C30—C39—C42	109.0 (6)
C13—C14—H14A	109.5	C39—C40—H40A	109.5
C13—C14—H14B	109.5	C39—C40—H40B	109.5
H14A—C14—H14B	109.5	H40A—C40—H40B	109.5
C13—C14—H14C	109.5	C39—C40—H40C	109.5
H14A—C14—H14C	109.5	H40A—C40—H40C	109.5
H14B—C14—H14C	109.5	H40B—C40—H40C	109.5
C13—C15—H15A	109.5	C39—C41—H41A	109.5
C13—C15—H15B	109.5	C39—C41—H41B	109.5
H15A—C15—H15B	109.5	H41A—C41—H41B	109.5
C13—C15—H15C	109.5	C39—C41—H41C	109.5
H15A—C15—H15C	109.5	H41A—C41—H41C	109.5
H15B—C15—H15C	109.5	H41B—C41—H41C	109.5
C13—C16—H16A	109.5	C39—C42—H42A	109.5
C13—C16—H16B	109.5	C39—C42—H42B	109.5
H16A—C16—H16B	109.5	H42A—C42—H42B	109.5
C13—C16—H16C	109.5	C39—C42—H42C	109.5
H16A—C16—H16C	109.5	H42A—C42—H42C	109.5
H16B—C16—H16C	109.5	H42B—C42—H42C	109.5
C19—C17—C20	107.6 (6)	C44—C43—C46	108.6 (8)
C19—C17—C18	108.5 (6)	C44—C43—C45	111.4 (8)
C20—C17—C18	110.0 (6)	C46—C43—C45	106.5 (7)
C19—C17—C10	110.2 (6)	C44—C43—C36	110.7 (6)
C20—C17—C10	112.6 (6)	C46—C43—C36	112.0 (6)
C18—C17—C10	107.9 (6)	C45—C43—C36	107.5 (6)
C17—C18—H18A	109.5	C43—C44—H44A	109.5
C17—C18—H18B	109.5	C43—C44—H44B	109.5
H18A—C18—H18B	109.5	H44A—C44—H44B	109.5
C17—C18—H18C	109.5	C43—C44—H44C	109.5
H18A—C18—H18C	109.5	H44A—C44—H44C	109.5
H18B—C18—H18C	109.5	H44B—C44—H44C	109.5
C17—C19—H19A	109.5	C43—C45—H45A	109.5
C17—C19—H19B	109.5	C43—C45—H45B	109.5
H19A—C19—H19B	109.5	H45A—C45—H45B	109.5
C17—C19—H19C	109.5	C43—C45—H45C	109.5
H19A—C19—H19C	109.5	H45A—C45—H45C	109.5
H19B—C19—H19C	109.5	H45B—C45—H45C	109.5
C17—C20—H20A	109.5	C43—C46—H46A	109.5
C17—C20—H20B	109.5	C43—C46—H46B	109.5
H20A—C20—H20B	109.5	H46A—C46—H46B	109.5
C17—C20—H20C	109.5	C43—C46—H46C	109.5
H20A—C20—H20C	109.5	H46A—C46—H46C	109.5
H20B—C20—H20C	109.5	H46B—C46—H46C	109.5
C26—C21—C22	120.0 (6)	C52—C47—N2	121.9 (6)
C26—C21—N1	120.6 (6)	C52—C47—C48	118.9 (6)
C22—C21—N1	119.3 (6)	N2—C47—C48	119.2 (7)
C21—C22—C23	120.5 (6)	C49—C48—C47	119.7 (7)
C21—C22—H22A	119.7	C49—C48—H48A	120.1
C23—C22—H22A	119.7	C47—C48—H48A	120.1
C24—C23—C22	117.8 (7)	C50—C49—C48	120.1 (6)
C24—C23—H23A	121.1	C50—C49—H49A	120.0
C22—C23—H23A	121.1	C48—C49—H49A	120.0
C23—C24—C25	121.7 (6)	C49—C50—C51	118.8 (6)
C23—C24—Br1	119.8 (5)	C49—C50—Br2	119.9 (5)
C25—C24—Br1	118.5 (5)	C51—C50—Br2	121.2 (6)
C24—C25—C26	119.7 (6)	C52—C51—C50	121.0 (7)
C24—C25—H25A	120.1	C52—C51—H51A	119.5
C26—C25—H25A	120.1	C50—C51—H51A	119.5
C21—C26—C25	120.2 (6)	C47—C52—C51	121.4 (6)
C21—C26—H26A	119.9	C47—C52—H52A	119.3
C25—C26—H26A	119.9	C51—C52—H52A	119.3
			
C8—N1—C1—C2	178.5 (7)	C47—N2—C27—C32	−169.1 (6)
C21—N1—C1—C2	−15.0 (11)	C34—N2—C27—C32	0.9 (7)
C8—N1—C1—C6	0.6 (7)	C47—N2—C27—C28	7.9 (10)
C21—N1—C1—C6	167.1 (6)	C34—N2—C27—C28	177.9 (6)
C6—C1—C2—C3	−0.5 (10)	C32—C27—C28—C29	0.0 (9)
N1—C1—C2—C3	−178.1 (6)	N2—C27—C28—C29	−176.7 (6)
C1—C2—C3—C4	−1.0 (10)	C27—C28—C29—C30	0.3 (10)
C2—C3—C4—C5	1.4 (10)	C28—C29—C30—C31	−0.7 (10)
C2—C3—C4—C13	−178.5 (6)	C28—C29—C30—C39	179.1 (6)
C3—C4—C5—C6	−0.2 (10)	C29—C30—C31—C32	0.9 (9)
C13—C4—C5—C6	179.7 (6)	C39—C30—C31—C32	−178.9 (6)
C4—C5—C6—C1	−1.1 (9)	N2—C27—C32—C31	177.5 (5)
C4—C5—C6—C7	177.4 (7)	C28—C27—C32—C31	0.2 (9)
C2—C1—C6—C5	1.5 (10)	N2—C27—C32—C33	−2.1 (7)
N1—C1—C6—C5	179.6 (5)	C28—C27—C32—C33	−179.4 (6)
C2—C1—C6—C7	−177.3 (6)	C30—C31—C32—C27	−0.7 (9)
N1—C1—C6—C7	0.7 (7)	C30—C31—C32—C33	178.8 (6)
C5—C6—C7—C9	1.3 (13)	C27—C32—C33—C34	2.6 (7)
C1—C6—C7—C9	179.9 (7)	C31—C32—C33—C34	−177.0 (7)
C5—C6—C7—C8	179.6 (7)	C27—C32—C33—C35	−177.6 (7)
C1—C6—C7—C8	−1.7 (7)	C31—C32—C33—C35	2.8 (12)
C9—C7—C8—C12	2.5 (10)	C35—C33—C34—C38	0.7 (10)
C6—C7—C8—C12	−176.1 (6)	C32—C33—C34—C38	−179.5 (6)
C9—C7—C8—N1	−179.2 (5)	C35—C33—C34—N2	178.0 (5)
C6—C7—C8—N1	2.1 (7)	C32—C33—C34—N2	−2.1 (7)
C1—N1—C8—C12	176.4 (7)	C27—N2—C34—C33	0.8 (7)
C21—N1—C8—C12	10.0 (11)	C47—N2—C34—C33	170.3 (6)
C1—N1—C8—C7	−1.7 (7)	C27—N2—C34—C38	177.9 (7)
C21—N1—C8—C7	−168.1 (6)	C47—N2—C34—C38	−12.6 (11)
C8—C7—C9—C10	−1.4 (9)	C34—C33—C35—C36	−2.4 (10)
C6—C7—C9—C10	176.8 (7)	C32—C33—C35—C36	177.8 (6)
C7—C9—C10—C11	0.0 (10)	C33—C35—C36—C37	1.4 (10)
C7—C9—C10—C17	177.3 (6)	C33—C35—C36—C43	−175.2 (6)
C9—C10—C11—C12	0.4 (11)	C35—C36—C37—C38	1.3 (11)
C17—C10—C11—C12	−176.9 (7)	C43—C36—C37—C38	177.9 (6)
C10—C11—C12—C8	0.6 (11)	C36—C37—C38—C34	−2.9 (11)
C7—C8—C12—C11	−2.1 (10)	C33—C34—C38—C37	2.0 (10)
N1—C8—C12—C11	−180.0 (6)	N2—C34—C38—C37	−174.9 (6)
C5—C4—C13—C16	−121.7 (7)	C31—C30—C39—C40	−120.9 (7)
C3—C4—C13—C16	58.2 (9)	C29—C30—C39—C40	59.3 (8)
C5—C4—C13—C14	−1.2 (9)	C31—C30—C39—C41	−2.6 (9)
C3—C4—C13—C14	178.7 (6)	C29—C30—C39—C41	177.6 (6)
C5—C4—C13—C15	118.2 (7)	C31—C30—C39—C42	118.2 (7)
C3—C4—C13—C15	−61.9 (8)	C29—C30—C39—C42	−61.6 (8)
C11—C10—C17—C19	−48.6 (9)	C35—C36—C43—C44	−142.2 (8)
C9—C10—C17—C19	134.1 (7)	C37—C36—C43—C44	41.3 (10)
C11—C10—C17—C20	−168.7 (7)	C35—C36—C43—C46	−20.8 (10)
C9—C10—C17—C20	14.0 (10)	C37—C36—C43—C46	162.7 (7)
C11—C10—C17—C18	69.7 (9)	C35—C36—C43—C45	95.9 (9)
C9—C10—C17—C18	−107.6 (7)	C37—C36—C43—C45	−80.6 (9)
C1—N1—C21—C26	−52.7 (9)	C27—N2—C47—C52	−58.5 (9)
C8—N1—C21—C26	111.3 (7)	C34—N2—C47—C52	133.7 (7)
C1—N1—C21—C22	129.1 (7)	C27—N2—C47—C48	120.4 (7)
C8—N1—C21—C22	−66.8 (9)	C34—N2—C47—C48	−47.4 (9)
C26—C21—C22—C23	0.0 (10)	C52—C47—C48—C49	3.2 (9)
N1—C21—C22—C23	178.2 (6)	N2—C47—C48—C49	−175.8 (6)
C21—C22—C23—C24	1.9 (10)	C47—C48—C49—C50	−0.5 (9)
C22—C23—C24—C25	−2.5 (10)	C48—C49—C50—C51	−1.4 (9)
C22—C23—C24—Br1	178.6 (5)	C48—C49—C50—Br2	174.4 (5)
C23—C24—C25—C26	1.1 (10)	C49—C50—C51—C52	0.7 (10)
Br1—C24—C25—C26	−179.9 (5)	Br2—C50—C51—C52	−175.1 (5)
C22—C21—C26—C25	−1.5 (10)	N2—C47—C52—C51	174.9 (6)
N1—C21—C26—C25	−179.6 (6)	C48—C47—C52—C51	−4.0 (10)
C24—C25—C26—C21	0.9 (10)	C50—C51—C52—C47	2.1 (10)

### Hydrogen-bond geometry (Å, °)

**Table:**

Cg3, Cg11 and Cg8 are the centroids of the C7–C12, C47–C52 and N2/C27/C32–C34 rings, respectively.

**Table:**

*D*—H···*A*	*D*—H	H···*A*	*D*···*A*	*D*—H···*A*
C22—H22A···Cg3^i^	0.93	2.75	3.544 (8)	144
C25—H25A···Cg11^ii^	0.93	2.92	3.511 (7)	123
C52—H52A···Cg8^iii^	0.93	2.95	3.591 (8)	127

Symmetry codes: (i) *x*+1, *y*, *z*; (ii) −*x*+1, −*y*+1, −*z*+1; (iii) *x*−1, *y*, *z*.

## References

[bb1] Allen, F. H., Kennard, O., Watson, D. G., Brammer, L., Orpen, A. G. & Taylor, R. (1987). *J. Chem. Soc. Perkin Trans. 2*, pp. S1–19.

[bb2] Enraf–Nonius (1985). *CAD-4 Software* Enraf–Nonius, Delft, The Netherlands.

[bb3] Harms, K. & Wocadlo, S. (1995). *XCAD4* University of Marburg, Germany.

[bb4] North, A. C. T., Phillips, D. C. & Mathews, F. S. (1968). *Acta Cryst.* A**24**, 351–359.

[bb5] Sheldrick, G. M. (2008). *Acta Cryst.* A**64**, 112–122. 10.1107/S0108767307043930 18156677

[bb6] Wang, C. S., Batsanov, A. S., West, K. & Bryce, M. R. (2008). *Org. Lett.* **14**, 3069–3072. 10.1021/ol801121p 18549228

[bb7] Weber, L. H. J., Boehling, L., Chrostowska, A., Dargelos, A., Stammler, H. G. & Neumann, B. (2011). *Eur. J. Inorg. Chem.* **20**, 3091–3101.

